# Genomic insights and survival dynamics of *Campylobacter* from ruminants in UHT milk, raw milk and dairy products

**DOI:** 10.3389/fmicb.2026.1791201

**Published:** 2026-03-11

**Authors:** Francesca Marotta, Roberta Di Romualdo, Elisa Di Domenico, Cecilia Villani, Emanuela Di Giulio, Anna Janowicz, Federica Di Timoteo, Giorgia Giorgini, Giusy Matteucci, Pierluigi Castelli, Laura Coccia, Simona Zoppi, Aurora Di Ilio, Cesare Cammà, Erica Tirloni, Simone Stella, Giuliano Garofolo

**Affiliations:** 1 National Reference Laboratory for Campylobacter, Istituto Zooprofilattico Sperimentale dell’Abruzzo e del Molise “G. Caporale”, Teramo, Italy; 2 National Reference Centre (NRC) for Whole Genome Sequencing of microbial pathogens: database and bioinformatics analysis (GENPAT), Istituto Zooprofilattico Sperimentale dell’Abruzzo e del Molise (IZSAM), Teramo, Italy; 3 Istituto Zooprofilattico Sperimentale del Piemonte, Liguria e Valle d’Aosta (IZS PLV) S.C. Diagnostica Generale, Turin, Italy; 4 Department of Veterinary Medicine and Animal Sciences, University of Milan, Lodi, Italy

**Keywords:** *Campylobacter*, dairy, food safely, ruminants, whole genome sequencing (WGS)

## Abstract

Broiler meat is the primary foodborne source of *Campylobacter*iosis; however, the discrepancy between reported human cases and recorded outbreaks worldwide suggests the need to investigate additional exposure sources. Milk is particularly relevant due to its association with outbreaks linked to raw milk consumption or inadequate pasteurization. Considering the role of ruminants as *Campylobacter* reservoirs, this study was undertaken to investigate *Campylobacter* prevalence on carcasses and intestinal contents of cattle and sheep sampled at slaughterhouses, as well as in raw milk and dairy products from farms and dairies. Whole-genome sequencing (WGS) was used to analyze the population structure, virulence, and antimicrobial resistance of 385 *C. jejuni* and *C. coli* isolates from cattle and sheep in Italy. Additionally, the survival of three *C. jejuni* strains was assessed in raw milk, UHT milk, and ricotta cheese under different temperatures and time points, using standard culture-based enumeration methods. Genome analysis revealed genetic diversity among ruminant isolates, with a pronounced host-specific structuring of *Campylobacter* populations. While CC206 and CC21 were the predominant clonal complexes overall, several lineages showed strict host association. CC658 was exclusively detected in sheep, whereas CC353, CC45, and CC49 were confined to cattle, indicating strong host-adapted patterns. Only two sequence types (ST1055 and ST10304) were shared between cattle and sheep, highlighting limited cross-host overlap and suggesting restricted interspecies transmission. 14 strains of animal and food origin exhibited a higher number of virulence-associated genes, while fewer than 10% showed multi-resistance to at least three antibiotic classes. Moreover, *C. jejuni* strains exhibited considerable resistance to refrigeration and dairy processing conditions, with distinct survival patterns depending on the dairy matrix. In particular, the *C. jejuni* 8264ST-403 complex exhibited enhanced persistence in ricotta cheese compared with UHT and raw milk, remaining viable throughout the entire experimental period and maintaining an average concentration of 4.21 log10 CFU/g at 42 days post-inoculation. This survival in ricotta underscores the ability of specific *C. jejuni* lineages to withstand adverse conditions in fresh dairy products, highlighting a potential public health risk. With an increasing consumer preference for raw dairy products, enhanced surveillance and control measures are essential to mitigate associated risks.

## Introduction

*Campylobacter*iosis is one of the most widespread foodborne illnesses globally, primarily caused by *Campylobacter jejuni*, which is responsible for an estimated nine million human cases annually in the European Union, leading to annual costs of healthcare estimated at about $1.9 billion (Kaakoush et al., 2015; European Food Safety Authority [EFSA], and European Centre for Disease Prevention and Control [ECDC], 2025). These zoonotic bacteria are frequent commensal components of the gut microbiota in birds and other animal species and can cause serious infections in humans. *Campylobacter*iosis symptoms include nausea, fever, abdominal pain, and severe diarrhea, with the potential to develop debilitating and sometimes fatal sequelae, especially in elderly or immunocompromised individuals (Kaakoush et al., 2015). Although antibiotic treatment is typically reserved for severe cases, antimicrobial-resistant *Campylobacter* spp. have emerged globally, potentially leading to treatment failure in both humans and animals. In particular, rising antimicrobial resistance to key antibiotics like macrolides and fluoroquinolones has been observed in *Campylobacter* strains from both animals and humans, posing a significant concern (Mohan et al., 2025). Multiple sources of infection have been identified, including animal feces, contaminated drinking water or raw milk, and particularly raw or undercooked meats (Arning et al., 2021). Poultry products remain the main source of human infection; however, there is growing evidence that ruminants represent the second largest reservoir (Sheppard et al., 2009; Roux et al., 2013; Ocejo et al., 2019). Globally, the consumption of raw milk is acknowledged as a significant public health concern and a recognized risk factor for *Campylobacter*iosis (Di Giannatale et al., 2016; Igwaran and Okoh, 2020; Sorgentone et al., 2021; Kenyon et al., 2020). Multiple epidemiological investigations have identified raw milk as the second most frequent vehicle of *Campylobacter* infection after poultry meat (Taghizadeh et al., 2022), and numerous outbreaks associated with the consumption of contaminated raw milk have been documented worldwide (Jaakkonen et al., 2020). According to EFSA, the incidence of *Campylobacter* in raw and pasteurized milk, as well as in dairy products including cheese, was 0.8% across Europe in 2022; however, some reporting countries showed higher percentages (2.6% Austria, 2.5% Slovenia and 1.1% Italy). In contrast, the incidence in meat and meat products from bovine and sheep was 1.2% across Europe and significantly higher in Italy (7.7%) in 2023. *Campylobacter* has been detected in sheep carcasses, retail lamb meat and liver, gallbladder, intestinal contents, and feces (Lazou et al., 2014a,b) and there is evidence of a relationship between strains isolated from sheep liver and those found in human diarrheal cases (Mughini Gras et al., 2012). Although contamination of red meat does not appear to pose a significant risk for human infection, *Campylobacter* strains present in ruminant feces can contaminate surface water and fresh products through agricultural runoff, accounting for an indirect transmission route (Clark et al., 2003; Mourkas et al., 2020). The rising consumer interest in natural and minimal processed foods has led to an increased consumption of raw and artisanal dairy products, sometimes without full awareness of the associated microbiological risks. Such alternatives may inadvertently elevate the risk of foodborne illnesses and subsequently increase healthcare expenditures. In several European countries, raw milk is directly marketed through vending machines outside dairy farms (Barata et al., 2022; Rapp et al., 2020). Therefore, the microbiological safety of these products must be ensured as the raw milk consumption raises a significant public health issue due to the possible contamination of this food with pathogens such as *Campylobacter*.

Despite its relevance, the presence of *Campylobacter* in ruminants has been less studied than in other food-producing animals and limited informations are available on its genetic diversity, virulence and antibiotic resistance patterns. This study was undertaken to collect new data on *Campylobacter* prevalence on carcasses and feces of cattle and sheep as well as in raw milk and dairy products. To better understand the role of ruminants in *Campylobacter* transmission, we used whole genome sequencing (WGS) to investigate genotyping, virulence and antimicrobial resistance profiles of *Campylobacter* isolates from cattle, sheep and dairy products. Moreover, given the known ability of several *C. jejuni* strains to survive in raw milk and induce illness in humans, a challenge test was performed to evaluate the survival and the impact of temperatures on the survival rates of different *C. jejuni* strains in different matrices such as raw milk, ultra-pasteurized (UHT) milk and ricotta cheese.

## Materials and methods

### Experimental design and strains selection

A randomized sampling was carried out in seven slaughterhouses across three regions of central Italy between 2020 and 2021. The selected facilities were key plants serving the main livestock production areas, ensuring broad geographic coverage of the regional production system. Cattle and sheep were sourced from 112 and 26 farms, respectively. The sampling set consisted of fecal material and carcass surface samples. A total of 79 raw milk samples were collected from seven bovine dairy farms, whereas 33 dairy products, including different types of cheeses, were obtained at retail from two dairy outlets. Both the farms and the two dairies were located in the same region of central Italy and were selected within the same region to ensure consistency and traceability of the local production chain. Other strains isolated from ruminants, available at the National Reference Laboratory (NRL) for *Campylobacter*^1^ were included in this study, for a total of 335 *C. jejuni* and 50 *C. coli* analyzed. 255 *C. jejuni* strains were isolated from cattle, including 194 from feces, 6 from carcasses, 1 from cheese, and 54 from milk. Additionally, 80 *C. jejuni* strains were isolated from sheep, with 73 from feces and 7 from carcasses. For *C. coli*, 45 strains were recovered from sheep: 21 from carcasses and 24 from feces. 5 strains were isolated from cattle: 4 from feces and 1 from a carcass. All strains were isolated during a 7-year period (2015-2022).

### MALDI-TOF identification and DNA extraction

The frozen stock preserved at –80°C in Microbank™ was cultured on Columbia blood agar plates at 42°C for 48 h in a microaerobic atmosphere (10% CO_2_, 5% O_2_ and balancing N_2_) in a controlled atmosphere incubator (ICO 240med, Memmert, Büchenbach, Germany). Following an initial phenotypic characterization, the colonies were confirmed to be *C. jejuni* or *C. coli* at the species level by using matrix-assisted laser desorption ionization–time of flight (MALDI-TOF) mass spectrometry (Bruker Daltonics GmbH, Bremen, Germany). Briefly, a portion of a colony from each isolate, extracted directly from the agar plate after 18–24 h of incubation to obtain fresh bacteria culture, was placed onto a 96-target plate in a single spot. Subsequently, one microliter of matrix solution (a saturated solution of cyano-4-hydroxycinnamic acid in 50% acetonitrile) was applied to the sample and allowed to crystallize by air-drying at room temperature for 5 min. The entire process, from MALDI-TOF mass spectrometry measurement to identification, was conducted automatically, requiring no user intervention. A score > 2.00 was attributed to each identification considered correct at species level. DNA extraction was performed using the QIAamp ^®^ DNA Mini Kit (Qiagen, Hilden, Germany) following the manufacturer’s recommendations. DNA concentrations were quantified using a Qubit™ 4 Fluorometer (Invitrogen, Carlsbad, CA) with dsDNA HS Assay Kit (Invitrogen) and a NanoDrop™ Lite Spectrophotometer (Thermo Fisher Scientific, Wilmington, DE, United States).

### Whole genome sequencing and genomic analysis

All *Campylobacter* isolates were sequenced. Briefly, total DNA was quantified using the Qubit dsDNA High Sensitivity Assay Kit on a Qubit Fluorometer (Thermo Fisher Scientific, Waltham, MA, United States). Sequencing libraries were prepared using the Illumina DNA Prep library preparation kit (Illumina, San Diego, CA, United States), following the recommended protocol. Library quality and fragment size distribution were assessed using an Agilent 4200 TapeStation (Agilent Technologies, Santa Clara, CA, United States) with High Sensitivity D5000 ScreenTape, and concentrations were normalized prior to pooling. The pooled libraries were sequenced on the Illumina NextSeq 500 sequencer and Illumina NextSeq 2000 platform using NextSeq500/550 cartridge (300 cycle) mid output kit and NextSeq 1,000/2,000 P1 cartridge (300 cycles) (Illumina, San Diego, CA, United States), respectively, generating paired-end reads of 150 bp. The sequence reads, consisting of 150-bp pair-end reads, were demultiplexed, the adapters were removed and trimmed using the Trimmomatic tool (version 0.36). *De novo* assembly was performed using SPAdes version 3.11.1 with the “careful” option selected. The sequence reads generated in this study were deposited in the NCBI Sequence Read Archive (SRA) under Bioprojects PRJNA1403798^2^ and PRJNA1403801.^3^ *C. jejuni* and *C. coli* genome assemblies were genotyped using both MLST and cgMLST.

The MLST profiles were assigned *ex novo* via MLST 7 loci schema v2.23.0, which scans contig files against PubMLST typing schemes^4^ (Jolley and Maiden, 2010). The cgMLST INNUENDO scheme with 678 loci (Llarena et al., 2018), implemented with chewBBACA software v2.8.5 (Silva et al., 2018; Zhou et al., 2018), was used to identify genomic clusters. The clusters of isolates were defined on the basis of cgMLST allele differences (Joseph et al., 2023). Minimum spanning trees (MSTreeV2) were calculated and visualized via ReportTree v2.5.3 with a partition threshold for clustering definition equal to 10 (“-thr = 10”) (Mixão et al., 2023). A Neighbor-Joining tree was constructed through pairwise analysis of identified alleles, with missing targets ignored using default settings. The resulting tree and associated metadata for *C. jejuni* were visualized using iTol v 6.7.2.

### Resistome and virulome characterization

The assemblies were analyzed for genomic antimicrobial resistance (AMR) traits and virulence genes. Antimicrobial resistance genes, specific genetic mutations known to confer resistance to fluoroquinolones (*gyrA*), macrolides (*23S rRNA*), and streptomycin (*rpsL*) and virulence genes were identified using Staramr v.0.8.0 and ABRicate v. 1.0.1^5^ by searching the publicly available Comprehensive Antibiotic Resistance Database (CARD) (2631 sequences, updated on 2023-Feb-21) (Jia et al., 2017; Zankari et al., 2017).

### Preparation of inoculum

*C. jejuni* strains 8264ST-403 complex, 2116ST-353 complex, and 883ST-21 complex were selected for the survival study as representative of genetically distinct and host-associated clonal complexes. These genotypes have been frequently associated with livestock reservoirs and human *Campylobacter*iosis, thereby providing epidemiologically relevant models to assess survival in dairy matrices. All strains were stored at the Italian National Reference Laboratory for *Campylobacter* (NRL, accessed on 20 June 2024).^6^ Strains were stored at –80°C in Microbank™. Bacteria were cultured on Columbia blood agar plates and incubated at 37°C for 24 h under microaerophilic conditions (10% CO_2_, 5% O_2_ and balancing N_2_), in a controlled atmosphere incubator (ICO 240med, Memmert, Büchenbach, Germany). After subculture on Columbia blood agar for 18 h under the same conditions, cells were suspended in Brain Heart Infusion (BHI) broth at OD600 = 0.3, corresponding to about 9 log_10_ CFU/ml (Krüger et al., 2014). Finally, a 10-fold dilution was performed to obtain a final concentration of 8 log_10_ CFU/ml, used to inoculate the raw milk, UHT milk and ricotta cheese.

### Inoculation of raw milk, UHT milk and ricotta cheese

UHT milk, raw milk samples collected from a regional milk producer in the Abruzzo region, and ricotta cheese samples purchased from a local supermarket, were spiked with the indicated *C. jejuni* strains to obtain an initial concentration of 8 log_10_ CFU/ml. UHT milk samples inoculated with *C. jejuni* 8264ST-403 complex, 2116ST-353 complex and 883ST-21 complex, were kept in closed 50-ml tubes and incubated at 4 ± 1°C and 25 ± 1°C for up to 42 days post-inoculation (d.p.i.) under normal atmospheric conditions. pH was measured (Mettler Toledo, Columbus, OH) throughout the experiment and remained stable at 6.76 ± 0.01 compared with the control samples at each temperature. Conversely, raw milk and ricotta cheese were inoculated with *C. jejuni* ST8264-403 complex. Raw milk samples were kept in closed 50-mL tubes, while ricotta cheese was inoculated through rubber septa and kept closed for the entire experiment. All samples were incubated at 4 ± 1°C for up to 42 days post-inoculation under normal atmospheric conditions. pH was measured in milk samples (Mettler Toledo, Columbus, OH) throughout the experiment and was stable at 6.75 ± 0.01 compared to control samples at each temperature. A 70 mL sample of raw milk was tested to assess its quality. The following parameters were evaluated: pH, somatic cell count, casein content, dry fat residue, lipid content, lactose levels, protein content, and mesophilic bacterial load.

## Statistical analysis

Data were analyzed using GraphPad Prism v. 10.4.1 software. For each sampling time, three independent experimental replicates were performed for each strain–matrix combination. The results are presented as mean values ± standard deviation (SD).

## Results

### Prevalence

A total of 285 animals (126 sheep and 159 cattle) were examined. Fecal samples and carcass swabs were collected from each animal. Among cattle, *Campylobacter* was detected in 34.6% of fecal samples (55/159), while only 3.8% (6/159) of carcasses tested positive for the bacterium. The predominant species isolated was *C. jejuni*, accounting for 81% of the isolates.

*Campylobacter* was detected in 42.1% of fecal samples (53/126) and in 27.8% of carcass swabs (35/126) in sheep, with *C. coli* as the predominant species isolated in 65% of the isolates.

Only 6.3% (5/79) of raw milk samples tested positive for *Campylobacter*, while all dairy products (Fresh cheese “Primo Sale,” Mozzarella “Bocconcini,” Caciotta cheese, Fior di latte cheese, Caciocavallo cheese, Bovine milk cheese, Cow’s milk butter, Yogurt) tested negative.

### MLST

**FIGURE 1:**
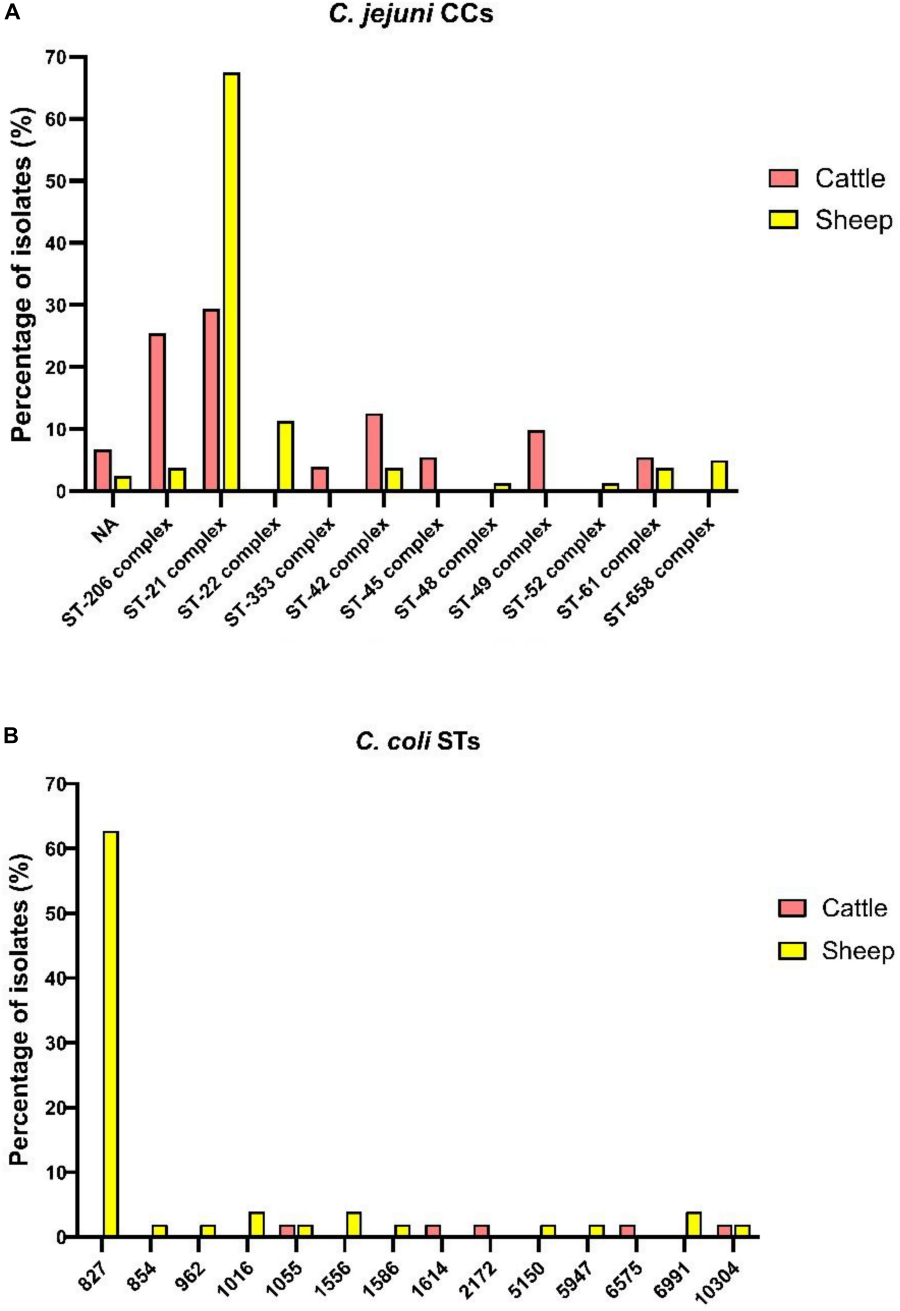
**(A)** *C. jejuni* CCs prevalence rates in ruminant species. **(B)** *C. coli* STs prevalence rates in ruminant species.

The sequence types (STs) and clonal complexes (CCs) of *C. jejuni* and *C. coli* found in the present study are presented in Figure 1. All *C. jejuni* isolates were categorized into 33 distinct STs. Depending on the host species, considerable genetic variation in *C. jejuni* strains was observed. Among these, 30 STs were attributed to 11 previously characterized clonal complexes, while the remaining 3 STs were not assigned to any CCs. The newly identified MLST profiles were integrated into the *Campylobacter* PubMLST database,^7^ as shown in Supplementary Tables 1, 2. Among *C. jejuni* isolates, CC206 and CC21 were predominant. Specifically, 67.5% (54/80) of the strains from sheep were assigned to CC21, whereas 29.4% (75/255) of the strains isolated from cattle were assigned to CC21 and an additional 25.5% (65/255) to CC206 (Figure 1A). Notably, the *C. jejuni* ST572 (CC206) and ST19 (CC21), were particularly prevalent, encompassing 76.5% (52/68) and 41.1% (53/129) of the isolates within their respective clonal complexes (Figure 1A).

The CC658 included isolates only from sheep; conversely, CC353, CC45, and CC49 included isolates only from cattle. The most frequently encountered clonal complexes in cattle isolates were CC21, CC206, CC42, CC49, CC61, and CC45, represented by 75, 65, 32, 25, and 14 isolates, respectively (Figure 1A). On the other hand, *C. coli* isolates were classified into 14 distinct sequence types indicating a high level of genetic diversity. Eleven of these STs belonged to a single clonal complex (CC828), representing 94% (47/50) of all *C. coli* isolates (Figure 1B). Among these, ST827 was the most prevalent in sheep, detected in 74.4% (32/43) of the samples. Notably, the only ST shared between sheep and cattle was ST10304. Interestingly, ST10304 was identified in the feces of both a sheep and a cow sampled at locations separated by a 2-h distance.

### cgMLST

**FIGURE 2:**
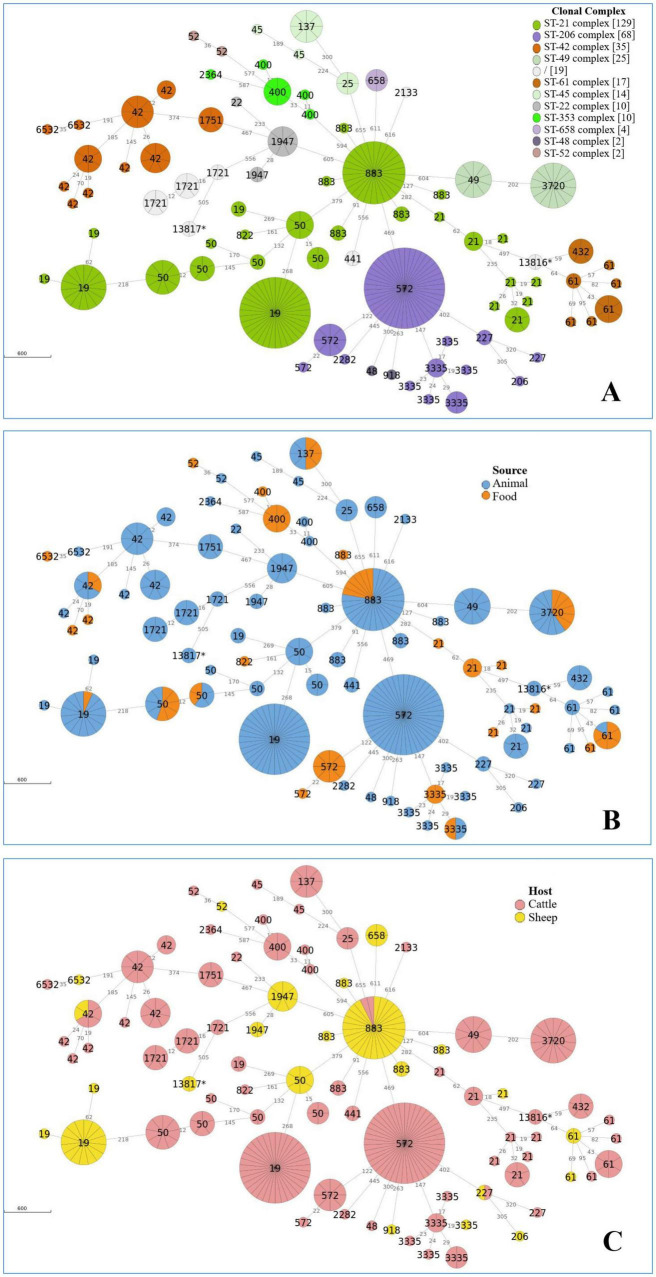
Minimum spanning tree (MST) calculated for 335 *C. jejuni* isolates from Italian ruminants, based on cgMLST profiles according to CCs **(A)**, source **(B)**, and host **(C).** The MST was generated with ReporTree (https://github.com/insapathogenomics/ReporTree). The circle size is proportional to the number of genomes for each cgMLST genotype. The branch labels correspond to the number of different alleles between cgMLST profiles. A cut-off of 10 allelic differences was applied to distinguish between several clusters. The numbers displayed within the circles represent Sequence Types (STs).

The cgMLST analysis of 335 *C. jejuni* genomes identified 43 distinct clusters using a clustering threshold of 10 allelic differences (Supplementary Table 3). Figure 2 presents the minimum spanning tree (MST), generated from cgMLST profiles of *C. jejuni* isolates. The MST is displayed according to clonal complexes (CCs) (Figure 2A), animal and food sources (Figure 2B), and host origin (cattle or sheep) (Figure 2C). Interestingly, among these, only three clusters, comprising STs 883, 227, and 42, were shared between the two-host species, while nine clusters, encompassing STs 50, 3720, 19, 3335, 137, 883, 61, and 42, included isolates from both food and animal sources (Supplementary Table 3 and Figures 2B,C). The largest cluster (Cluster 7) comprised 43 *C. jejuni* isolates belonging to ST572, all originating from bovine faces (Figure 2C). These isolates differed by 122 alleles from 8 *C. jejuni* ST572 strains isolated from raw milk (Figure 2B). The second-largest cluster (Cluster 8) contained 34 *C. jejuni* strains of ST19 from bovine faces, which differed by at least 274 alleles from 15 *C. jejuni* ST19 strains isolated from sheep (Figure 2C). The third-largest cluster (Cluster 6) included 27 *C. jejuni* ST883 strains derived from food and sheep faces (Figure 2B). Notably, this cluster also contained one strain isolated from cattle feces and one from cheese (Figure 2B). These isolates were collected over a 3-year period (2017–2020). Another cluster (Cluster 9) comprised 15 *C. jejuni* ST3720 isolates, including nine recovered from cattle faces and 6 from raw milk (Figure 2B). In contrast, the 10 strains grouped in the final large cluster (Cluster 2, ST49) were exclusively of bovine origin (Supplementary Table 3 and Figure 2C).

**FIGURE 3:**
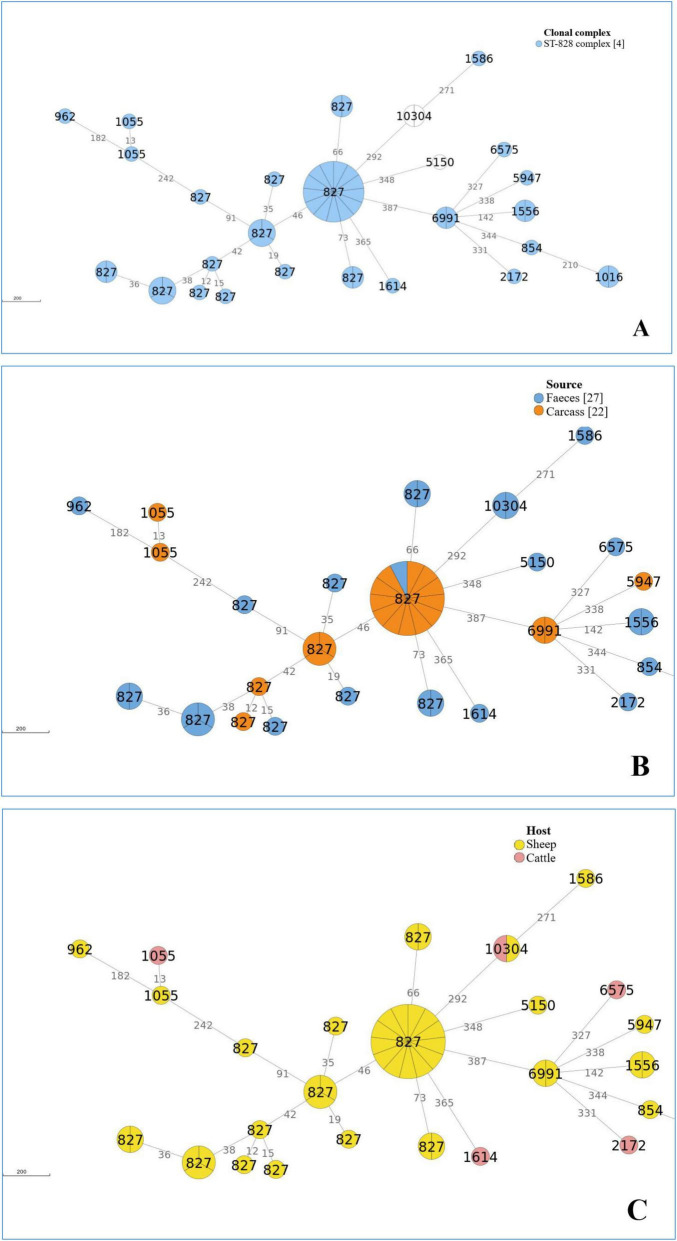
Minimum spanning tree (MST) calculated for 50 *C. coli* isolates from Italian ruminants, based on cgMLST profiles according to CCs **(A)**, source **(B)** and host **(C).** The MST was generated with ReporTree (https://github.com/insapathogenomics/ReporTree). The circle size is proportional to the number of geomes for each cgMLST genotype. The branch labels correspond to the number of different alleles between cgMLST profiles. A cut-off of 10 allelic differences was applied to distinguish between several clusters. The numbers displayed within the circles represent Sequence Types (STs).

The cgMLST analysis of 50 *C. coli* genomes identified 10 distinct clusters using a clustering threshold of 10 allelic differences (Supplementary Table 4). Figure 3 presents the minimum spanning tree (MST), generated from cgMLST profiles of *C. coli* isolates. The MST is displayed according to clonal complexes (CCs) (Figure 3A), animal and food sources (Figure 3B), and host origin (cattle or sheep) (Figure 3C).

The largest cluster (Cluster 1) comprised 13 *C. coli* isolates belonging to 827ST-828 complex, all originating from sheep faces (Supplementary Table 4 and Figure 3C).

### Detection of antimicrobial resistance genes

**FIGURE 4:**
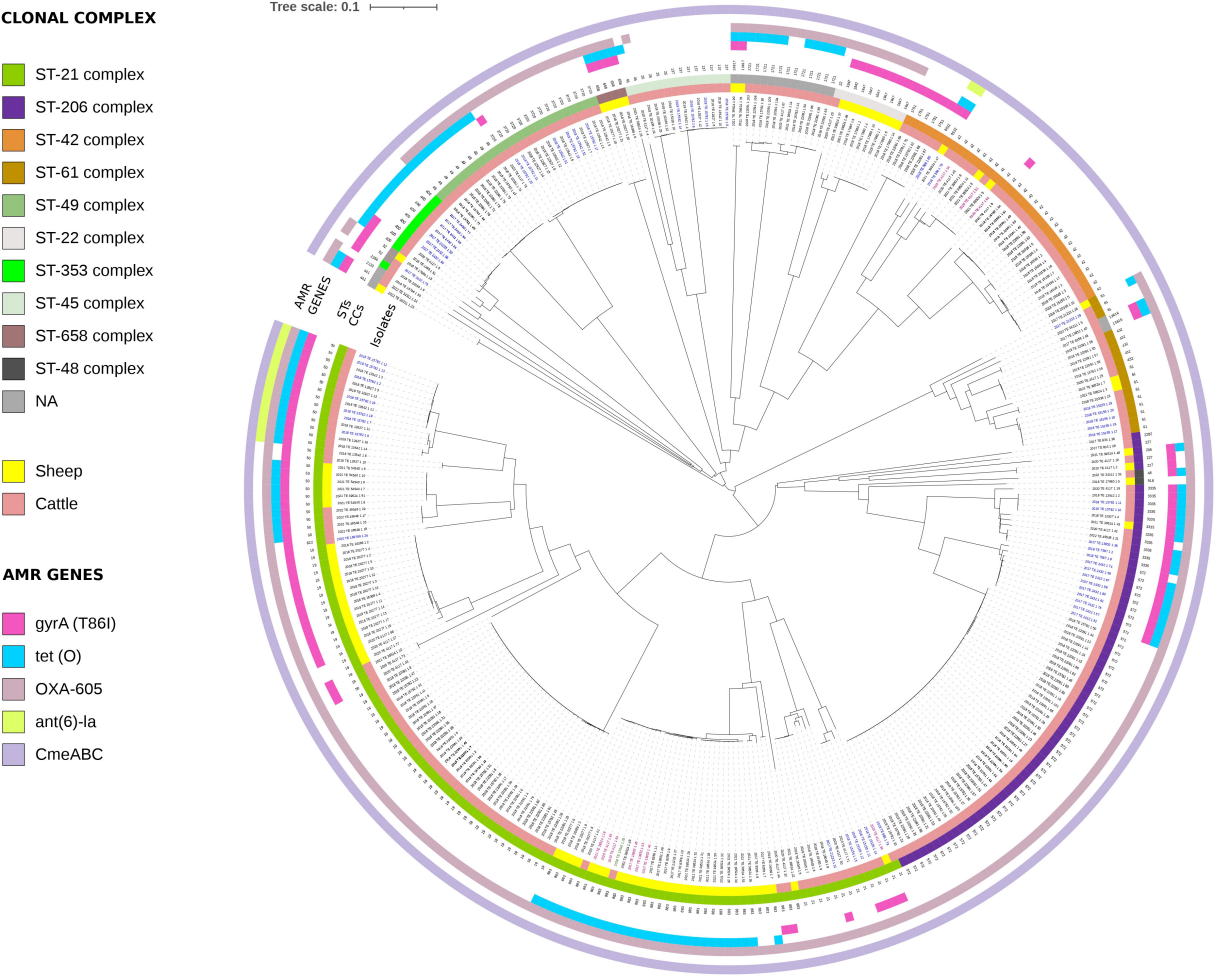
Phylogenetic analysis of *C. jejuni* strains from ruminants based on cgMLST analysis. CCs are shown in colored ring for each strain, while STs are indicated with numbers. The isolates noted in blue indicate *C. jejuni* from raw milk, those noted in red denote *C. jejuni* from carcasses, those highlighted in green represent *C. jejuni* from cheese, and those in black correspond to *C. jejuni* from feces. The presence of AMR genes is denoted by colored circles: *gyrA*_T86I (fuchsia), *tetO* (light blue), *oxa*-605 (purple), *ant(6)-la* (green), and i (gray).

*C. jejuni* WGS analysis detected 6 AMR genes and one-point mutation associated with antibiotic resistance to quinolones. Distribution of genetic determinants of resistance identified by WGS in each isolate is shown in Supplementary Table 1 and in Figure 4 along with the corresponding MLST profile (ST and CC). The *bla_*OXA*–605_* gene encoding resistance to β-lactams was the most commonly detected resistance gene, present on average in 82.7% of isolates (277/335), except for CC42, CC353 and CC45 strains. The prevalence of the *tetO* gene (34.6%) was highest among isolates from CC658 (100%) and CC353 (90%), with subsequent occurrences in isolates from CC21 (41.1%), CC49 (40%), and CC206 (27.9%). Resistance to quinolones was encoded in all cases by a chromosomal point mutation in the *gyrA* gene (C257T; T86I) present in 32.5% of the isolates from CC658 (100%), CC48 (50%), CC206 (33.8%), CC353 (30%), and CC21 (40.3%). The *cmeABC* operon, responsible for encoding a multidrug efflux pump and comprising the *cmeA*, *cmeB*, and *cmeC* genes, was found in 100% of *C. jejuni* isolates. Finally, the *ant(6)-Ia* gene, which confers resistance to streptomycin, was detected in 4.7% of strains, particularly prevalent among those belonging to CC21. Similarly, 82% of *C. coli* isolates exhibited resistance to β-lactams, while 72% were resistant to tetracycline. The *cmeA, cmeB*, and *cmeC* genes were differentially distributed among *C. coli* isolates: *cmeA* was detected in only 12% of the isolates, whereas *cmeB* and *cmeC* were present in 98 and 100%, respectively. Resistance to quinolones was consistently associated with a chromosomal point mutation in the *gyrA* gene (C257T; T86I), found in 44% of the isolates. Finally, 14% of *C. coli* isolates carried the *aadE-Cc* gene, responsible for streptomycin resistance. Supplementary Table 2 lists the genetic AMR determinants investigated in this study for *C. coli*.

### Detection of virulence factors

The analysis of the virulome revealed the presence of 111 virulence genes among 335 *C. jejuni* isolates. These genes were categorized into eight distinct groups according to their functions in pathogenesis and colonization, as outlined in Supplementary Table 1 and Supplementary Figure 1. In the present study, our *C. jejuni* collection was divided in three main clusters, according to the prevalence of investigated virulence genes.

Cluster 1 included 10 clonal complexes (CC353, 49, 658, 45, 22, 42, 61, 206, 52, and 48), respectively present in 30% (24/80) and in 63.9% (163/255) of sheep and bovine isolates. This cluster was characterized by the presence of about half of the immune evasion LOS and capsule biosynthesis genes investigated; conversely, almost all toxin, motility, adhesion, chemotaxis, invasion and capsule production genes were present. Cluster 2 comprised only one clonal complex (CC21 with ST21 and ST883) isolated in 38.8 (31/80) and 6.7% (17/255) of sheep and bovine, respectively. This cluster grouped all of the virulome genes with prevalence ranging from 70% up to 100%. Similarly, Cluster 3 included one clonal complex (CC21 with ST19, ST50 and ST822) isolated from 28.8% (23/80) to 22.7% (58/255) of sheep and bovine. Almost all of the virulence genes responsible for immune evasion, including those related to LOS, toxin production, motility, adhesion, chemotaxis, invasion, and capsule production, mainly formed the cluster. In contrast, capsule biosynthesis genes were present in only 40% of the investigated isolates.

Only 23 virulence genes were identified in *C. coli* isolates, primarily associated with motility, adhesion, chemotaxis, and immune evasion LOS. Motility-related genes were the most prevalent (*flaG*, *fliS*, *pseE/maf5*, *ptmA*, *ptmB*), detected in approximately 80% of the isolates, followed by the *fliD* gene, which plays a role in chemotaxis, and the *flgM* gene, involved in adhesion. The only gene associated with immune evasion LOS was *waaF*, which was present in 10% of the isolates. The virulence genes detected for *C. coli* are listed in Supplementary Table 2. Overall, the distinct virulence gene compositions observed among the three clusters suggest different pathogenic and adaptive strategies within ruminant-associated *Campylobacter* populations. The broad distribution of genes involved in motility, adhesion, invasion, and toxin production in Cluster 1 may reflect a generalist virulence profile, consistent with its association with multiple clonal complexes and both ruminant hosts. In contrast, the high prevalence of virulence determinants in Clusters 2 and 3, both associated with CC21 lineages frequently implicated in human *Campylobacter*iosis, suggests a potentially enhanced pathogenic capacity and a closer link to clinically relevant strains. The differential presence of capsule biosynthesis genes among clusters may further indicate variation in immune evasion strategies and host adaptation. Together, these patterns align with previously described host-associated and pathogenic lineages and support the relevance of virulence clustering as a tool to infer potential differences in pathogenicity and ecological specialization among *Campylobacter* isolates.

### Survival of *Campylobacter* in UHT milk, raw milk and cheese ricotta at different temperatures

**FIGURE 5:**
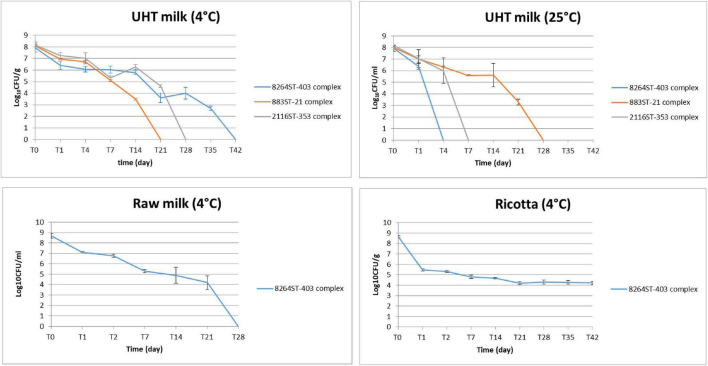
Survival curves of *C. jejuni* in UHT milk, raw milk and ricotta cheese. Three *C. jejuni* strains were inoculated at an initial concentration of approximately 8 log_10_ CFU/ml in UHT milk, raw milk and ricotta cheese and stored aerobically at refrigeration (4 ± 1°C) and ambient temperatures (25± 1°C). Samples were collected periodically to assess bacterial survival by CFU count. Blue line, *C. jejuni* 8264ST-403 complex; orange line, *C. jejuni* 883ST-21complex; gray line, *C. jejuni* 2116ST-353 complex. The *x*-axis shows time (days post-inoculation) and the *y*-axis shows viable count (log_10_ CFU/mL or g). Data represent the mean bacterial count ± standard deviations from three independent replicate experiments.

The survival of three distinct *C. jejuni* strains was investigated in UHT milk stored aerobically at refrigeration (4 ± 1°C) and ambient temperature (25 ± 1°C) (Figure 5). The CFU decreased over time in a strain-dependent manner. In detail, the *C. jejuni* 8264ST-403 complex and the CFU of *C. jejuni* outbreak strain 883ST-21 complex declined most slowly, respectively, at 4°C and 25°C in UHT milk, in comparison to *C. jejuni* 2116ST-353 complex. In particular, *C. jejuni* 8264ST-403 complex and *C. jejuni* 883ST-21 complex were recovered until at 35 days and 28 days at refrigeration temperature, respectively. The survival dynamic at 25°C in UHT milk showed a shorter period of viability for the *C. jejuni* 2116ST-353 complex and the 8264ST-403 complex which were detected only after no more than 4 days and 1 day, respectively. On the contrary the *C. jejuni* outbreak strain 883ST-21 complex survived for at least 21 days.

Additionally, the survival of *C. jejuni* 8264ST-403 complex was also assessed in raw milk and ricotta cheese, both stored at 4 ± 1°C. The *C. jejuni* 8264ST-403 complex survived for a shorter period in raw milk, being detectable until 21 post-inoculation days, which shortened the survival period by 2 weeks compared with UHT milk. The compositional analysis of raw milk samples from T 0 to T 17 days post-inoculation is shown in Table 1. Raw milk samples exhibited comparable values of pH, casein, solids-no-fat, lactose and protein. The total bacterial count in raw milk ranged from 12,000 CFU/ml at T 0 to 740,000 CFU/ml at T 17.

**TABLE 1:** Composition and total bacterial count of raw milk samples.

Time	pH	Somatic cells (× 1,000/mL)	Casein (% w/v)	Solids-not-fat (% w/v)	Fat (% w/v)	Lactose (% w/v)	Protein (% w/v)	Total bacterial count (× 1,000/CFU per mL)
T 0	6.76	570	2.64	9.12	4.18	5.02	3.46	12
T 24 h	6.75	468	2.48	8.76	3.65	4.69	3.42	14
T 48 h	6.75	444	2.57	8.78	3.41	4.60	3.45	13
T 17 d.p.i.	6.75	285	2.52	8.75	3.39	4.58	3.45	740

The survival kinetics of the *C. jejuni* 8264ST-403 complex in ricotta cheese differed markedly from those observed in both UHT and raw milk, as the strain remained viable throughout the entire experimental period, maintaining an average concentration of 4.21 log_10_ CFU/g at 42 days post-inoculation.

## Discussion

This study offers valuable insights into the prevalence, genetic diversity, antimicrobial resistance and virulence factors of *C. jejuni* and *C. coli* strains isolated from cattle and sheep in Italy, with a focus on their potential role in the contamination of raw milk and dairy products and the associated risks to human health. Our findings highlight that in Italy both cattle and sheep are significantly colonized by *Campylobacter* spp., suggesting the important role of reservoirs of *Campylobacter*iosis within the national epidemiological framework. In cattle, *Campylobacter* was detected almost exclusively in fecal samples, with low prevalence on carcasses (Knipper et al., 2022). In contrast, in sheep, the bacterium was found at considerable levels in both matrices examined. The predominant species identified were *C. jejuni* in cattle and *C. coli* in sheep, consistent with observations reported by other authors (Sánchez et al., 2019).

Genotyping of the *Campylobacter* spp. isolates from cattle and sheep in Italy revealed a high genetic diversity. CC21 was the largest and most widely distributed clonal complex, predominantly prevalent in sheep, followed by CC206, which was mainly detected in cattle.

The predominance of CC21 was not unexpected, since it is known as a host-generalist clonal complex, and it was reported previously in other studies on ruminants (Thépault et al., 2018; Aksomaitiene et al., 2019; Garcia-Fernandez et al., 2024). Surprisingly, only three STs (883, 227, and 42) were found to be shared between both animal species, confirming a host species-specific separation, as previously demonstrated also by other authors (Ocejo et al., 2021). Moreover, the clustering analysis revealed that *C. jejuni* isolates from animals and food of animal origin products were assigned to five distinct clusters comprising the STs 883, 42, 3720, 61, and 400, indicating a potential transmission route of *C. jejuni* strains between animals and foods, specifically raw milk. The identification of the CC658 exclusively associated with sheep highlights the specificity of certain *C. jejuni* lineages to particular hosts. Conversely, the broader diversity observed in cattle isolates suggests that cattle may serve as a more heterogeneous source of *C. jejuni*, contributing to the genetic complexity of potential infection sources. In addition, WGS data analysis revealed that *C. jejuni* lineages from ruminants in our dataset appeared to differ from those reported as prevalent in poultry (Marotta et al., 2023), suggesting that domestic animals (cattle and chicken) are genetically distant, even if it was known that phylogenetically distant animals can harbor closely related strains, especially in wild populations (Sheppard et al., 2011). In terms of antimicrobial resistance, approximately 10% of *C. jejuni* strains exhibited resistance to multiple classes of antibiotics. The presence of resistance genes such as *blaOXA-605* (β-lactams) and *tetO* (tetracyclines) in a large proportion of isolates, particularly those from sheep, raises concerns about the efficacy of commonly used antibiotics in the treatment of *Campylobacter*iosis (Marotta et al., 2019). The detection of quinolone resistance, mediated by a point mutation in the *gyrA* gene, was also observed, particularly among strains from CC658 and CC353. Fortunately, the prevalence of multidrug resistant genotypes was very low (<10%). However, these findings highlight the evolving challenge of antimicrobial resistance in *C. jejuni* from ruminants and the need for careful monitoring and prudent antibiotic use in both agricultural and clinical settings (Wang et al., 2023; Wang et al., 2025; Jia et al., 2025; Yang et al., 2025).

The significant public health risks posed by *C. jejuni* are due to specific virulence genes that constitute its virulome. This study revealed a large number of *C. jejuni* genomes characterized by a notable presence of virulence genes, categorized into three main clusters. The major differences among *C. jejuni* strains were observed for two classes of genes involved in lipooligosaccharide synthesis (group 1) and capsule biosynthesis (group 2), which we have previously identified as the most variable also among different STs of *C. jejuni* isolated from humans (Garcia-Fernandez et al., 2024). Several genes of group 1 (*cysC*, *gmhA*, *gmhA2*, *gmhB*, *hddA*, *hddC*, *waaC*, *waaF*, *waaV*, *wlaN*) and capsular polysaccharide (CPS) genes involved in capsule biosynthesis (*Cj1416c*, *Cj1417c*, *Cj1418c*, *Cj1419c*, *Cj1420c*, *kps C-D-E-F*) in addition to genes related to toxin production, motility, adhesion, chemotaxis and invasion genes, were present in almost all isolates.

All *C. jejuni* isolates were characterized by the presence of the *wlaN* gene, which encodes the production of Beta-1,3 galactosyltransferase involved in cell wall synthesis and cytotoxin production, as well as the cytolethal distending toxin genes *cdtA*, *cdtB*, and *cdtC* (Koga et al., 2006). The product of the *wlaN* gene also exhibits ganglioside-mimicking structures, which are believed to play a role in the development of Guillain–Barré syndrome following *C. jejuni* infection (Guirado et al., 2020; Datta et al., 2003). In particular, *C. jejuni* strains from Cluster 2, and especially those from Cluster 3, were found to carry the highest number of virulence factors including genes associated with other important steps of infection such as the *cdt* toxin gene and most of the flagellar biosynthesis and chemotaxis-related genes described for *C. jejuni.* These findings suggest a greater virulence potential, reflecting different adaptive and pathogenic strategies among ruminant-associated *Campylobacter* populations; however, proteins presumably involved in bacterial adherence, invasion of epithelial cells and/or toxin production, were not assessed in this study. Nevertheless, the high prevalence of these genes in our genome collection underscores the importance of these genes for *C. jejuni* vitality. Our results are in line with those of Panzenhagen et al., who suggested that the high prevalence of the *kpsM-S-T* locus genes may support further exploration of their possible role in future vaccine-oriented studies (Panzenhagen et al., 2021). Notably, *C. coli* ST10304 strains harbored the highest number of virulence determinants, potentially enhancing their evolutionary adaptability. Several studies have reported different CCs prevalence rates in cattle, sheep, and goats (Rivas et al., 2021; Ocejo et al., 2021).

In our study, the impact of *Campylobacter* on the contamination of fresh beef carcasses appears to be limited compared to sheep. This difference may be related to slaughtering hygiene and the size of the animals, as smaller carcasses are more susceptible to fecal contamination during slaughtering processes than larger ones. Conversely, *Campylobacter* detection in milk was relatively infrequent and likely associated with inadequate milking hygiene or, in rare cases, mastitis caused by *Campylobacter* spp. (Anand et al., 2015; Ito Eleodoro et al., 2023). Nevertheless, the identification of a few positive samples underscores the importance of proper milk hygiene and consequently it is important to adopt proper sanitation practices. A separate consideration concerns dairy products which have not been detected contaminated by *Campylobacter*. The presence of *Campylobacter* spp. in dairy products, particularly those with long maturing periods, is seldom reported (El-Zamkan and Hameed, 2016). The role of this category of products remains controversial and, in some cases, they have been identified as sources of *Campylobacter* outbreaks (Sorgentone et al., 2021). In this study, challenge tests demonstrated that the selected *C. jejuni* strains were able to survive in fresh and minimally processed dairy matrices, including ricotta cheese, UHT milk, and raw milk, particularly under refrigeration conditions. The inoculum concentration used in the experiments exceeds levels typically reported in naturally contaminated dairy products; however, this approach was intentionally adopted to enable controlled evaluation of comparative survival dynamics rather than to reproduce field conditions. Moreover, as the experiments were conducted using a limited number of genetically characterized strains, the findings cannot be generalized to the entire *C. jejuni* population. Nevertheless, the observed persistence of certain genotypes at low temperatures indicates that some strains may retain viability for extended periods in selected dairy matrices. Overall, these results highlight the potential for strain-dependent persistence in dairy environments, while further studies including a broader diversity of isolates are required to confirm and extend these observations.

This finding is particularly relevant in the context of growing consumer preference for raw milk and artisanal dairy products, which could be associated with a higher risk of exposure to *Campylobacter.*

Moreover, the ability of *Campylobacter* to survive in the ricotta cheese throughout the entire testing period under the conditions applied in this study suggests that the bacterium may remain viable for prolonged durations, underscoring the potential public health risk posed by its persistence in products commonly perceived as low risk when stored at low temperatures. The survival of these bacteria in dairy products could suggest the importance of continued surveillance, the need for improved surveillance, especially in regions where raw milk and unpasteurized dairy products are widely consumed. The notably high prevalence (26%) of ST883-21 complex observed in our study, together with the detection of strains from both animals and dairy-derived food products (cheese) within the same cluster, is noteworthy. In the outbreak investigation reported by Sorgentone et al. (2021), 60 laboratory-confirmed human cases were identified, and *C. jejuni* was isolated both from patients and from caciotta cheese, with wgMLST analysis demonstrating that all isolates belonged to the same genomic cluster, thereby confirming the dairy product as the infection vehicle.

Furthermore, follow-up investigations of milk-borne outbreaks have documented persistent contamination of bulk tank milk by ST883 strains for at least 7 months, and experimental survival assays showed that ST883 strains survived 4–6 days in refrigerated raw milk at an inoculum of 105 CFU/ml, compared to ≤ 3 days for other sequence types under identical conditions (Bianchini et al., 2014). Similarly, Jaakkonen et al. (2019) reported that ST883 strains were detected on a dairy farm for at least 11 months, whereas other sequence types were recovered only sporadically.

Taken together, our study reports a 26% prevalence of the ST883-21 complex among the analyzed ruminant and dairy isolates, while outbreak investigations have documented prolonged environmental persistence of this lineage and its confirmed involvement in human cases. These complementary observations could provide quantitative context supporting the potential relevance of ST883 in dairy-associated settings. Accordingly, strengthened control measures and continued consumer awareness regarding raw milk consumption may help reduce possible public health exposure to *C. jejuni*.

However, several limitations of the present study should be acknowledged. Animal sampling was geographically restricted to three regions of central Italy and conducted in seven slaughterhouses, which may limit the generalizability of these findings to different geographic settings or production systems. Including isolates collected over multiple years, allows for a broader representation of *Campylobacter* genetic diversity in ruminants. While some temporal variation may exist, the study focuses on overall genomic diversity and epidemiologically relevant lineages rather than short-term trends, reducing the impact of potential temporal bias.

Survival experiments were conducted with a limited number of selected *C. jejuni* strains; however, this targeted strain selection does not capture the full genetic diversity and may introduce some bias, it enabled the use of well-characterized, biologically relevant genotypes to assess survival in dairy matrices. Moreover, despite the relatively low proportion of *Campylobacter*-positive raw milk samples, the controlled survival assays, provide key evidence of the potential for *C. jejuni* to persist in dairy environments. Future investigations including a broader geographic coverage, larger sample sizes, temporal sampling and a greater genetic diversity are needed to clarify the role of ruminants and dairy products in *Campylobacter* transmission. Our findings seem suggest the potential persistence of this lineage in livestock and dairy environments, although further epidemiological analyses are required to define its impact on human infection.

## Conclusion

Monitoring antimicrobial resistance and conducting genetic epidemiology studies can enhance the tracking of *Campylobacter* spp. occurrence and spread in ruminant populations, provide deeper insights into the relationships among isolates, and improve the assessment of potential transmission risks to humans via the food chain and environmental contamination. The implementation of stringent hygiene protocols on dairy farms, including meticulous manure management, rigorous feed hygiene, adherence to effective milk pasteurization standards, as well as storage time control and cold-chain management for fresh dairy products, should be considered as part of integrated risk management strategies to mitigate the risk of *Campylobacter c*ontamination and reduce possible public health exposure. Our findings underscore the value of incorporating WGS into coordinated surveillance programs involving industry stakeholders, regulatory authorities, and public health agencies. Such an integrated approach may generate comprehensive insights into *Campylobacter* genetic diversity and enhance the capacity to trace potential sources of infections that might otherwise appear sporadic.

## Data Availability

The datasets presented in this study can be found in online repositories. The names of the repository/repositories and accession number(s) can be found in the article/Supplementary material.

## Footnotes

1
http://www.izs.it/IZS/Eccellenza/Centri_nazionali/LNR_-_Campylobacter

2
https://www.ncbi.nlm.nih.gov/bioproject/PRJNA1403798

3
https://www.ncbi.nlm.nih.gov/bioproject/PRJNA1403801

4
https://pubmlst.org

5
https://github.com/tseemann/abricate/

6
http://www.izs.it/IZS/Eccellenza/Centri_nazionali/LNR_-_Campylobacter

7
https://pubmlst.org/organisms/Campylobacter-jejunicoli/
