# Supplementary material for: Genomic insights and survival dynamics of Campylobacter from ruminants in UHT milk, raw milk and dairy products
Source: Front Microbiol. 2026 Mar 11;17:1791201. doi: 10.3389/fmicb.2026.1791201 (PMC13013509; doi:10.3389/fmicb.2026.1791201)
Supplement: Supplementary file 1 [file Data_Sheet_1.pdf]

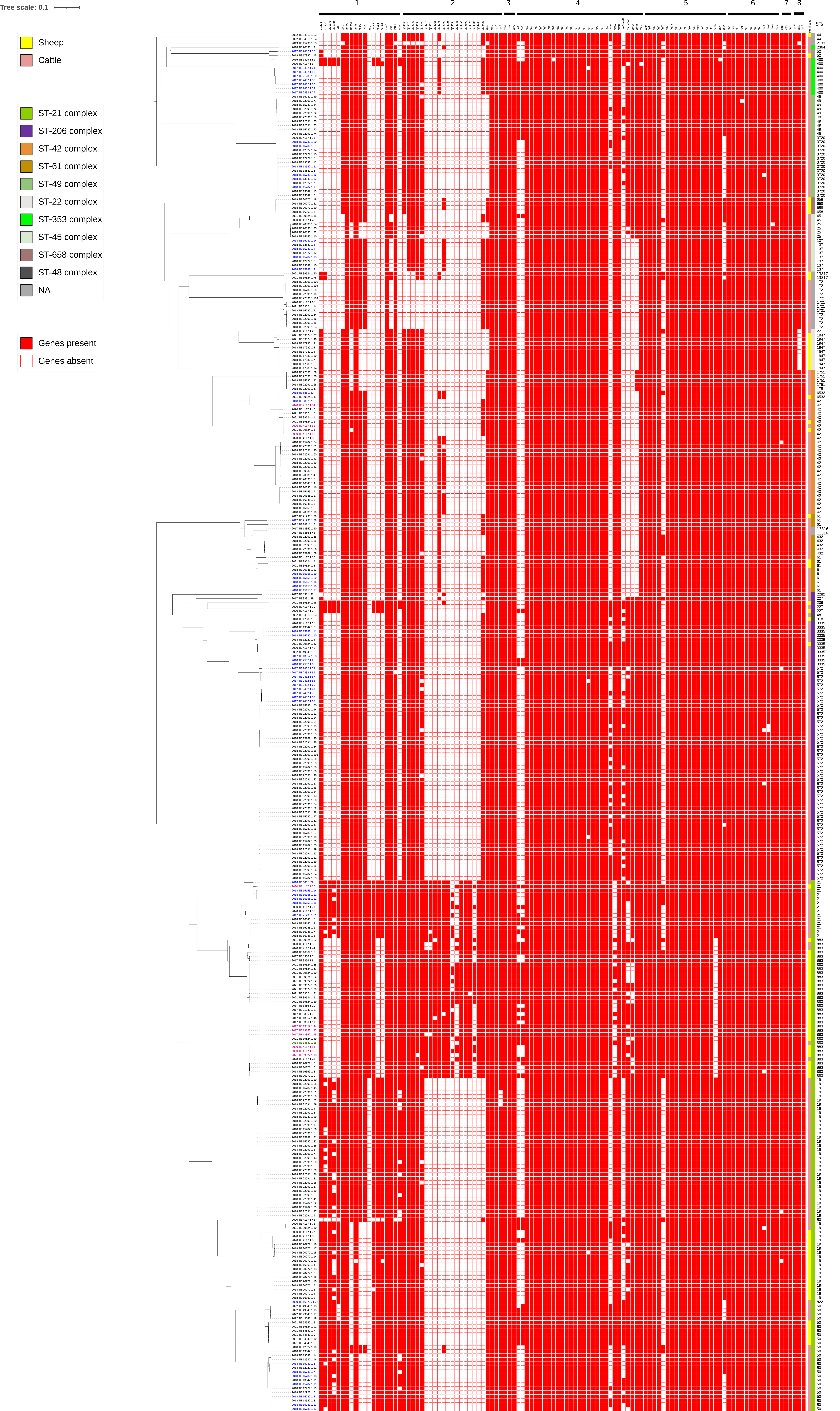
Tree scale: 0.1
Sheep
Cattle
ST-21 complex
ST-206 complex
ST-42 complex
ST-61 complex
ST-49 complex
ST-22 complex
ST-353 complex
ST-45 complex
ST-658 complex
ST-48 complex
NA
Genes present
Genes absent
1
2
3
4
5
6
7
8
STs

Figure S1. Virulome analysis of *C. jejuni* strains isolated from ruminants. Virulence determinants were divided into eight different categories indicated by numbers 1-8: 1) immune evasion LOS; 2) capsule biosynthesis; 3) toxin; 4) motility 5) adhesion; 6) chemotaxis; 7) invasion; 8) capsule production. The colour strips of Y-axis represent CCs corresponding to each strain. The reported numbers of X-axis represent the categories of investigated genes. Binary heat maps show the presence and absence of virulence genes. Coloured cells represent the presence of genes. The isolates noted in blue indicate *C. jejuni* from raw milk, those noted in red denote *C. jejuni* from carcasses, those highlighted in green represent *C. jejuni* from cheese, and those in black correspond to *C. jejuni* from faeces.
